# Cytoplasmic chromatin fragments—from mechanisms to therapeutic potential

**DOI:** 10.7554/eLife.63728

**Published:** 2021-01-29

**Authors:** Karl N Miller, Nirmalya Dasgupta, Tianhui Liu, Peter D Adams, Maria Grazia Vizioli

**Affiliations:** 1 Tumor Initiation and Maintenance Program, Sanford Burnham Prebys Medical Discovery InstituteLa JollaUnited States; 2 Cancer Research United Kingdom Beatson Institute, Garscube EstateGlasgowUnited Kingdom; 3 Institute of Cancer Sciences, University of Glasgow, Garscube EstateGlasgowUnited Kingdom; 4 Department of Physiology and Biomedical Engineering, Mayo ClinicRochesterUnited States; 5 Robert and Arlene Kogod Center on Aging, Mayo ClinicRochesterUnited States; Baylor College of MedicineUnited States; Weill Cornell MedicineUnited States

**Keywords:** aging, senescence, mitochondria, cytoplasmic chromatin fragments, epigenetics

## Abstract

Senescent cells, damaged cells that permanently exit the cell cycle, play important roles in development, tissue homeostasis, and tumorigenesis. Although many of these roles are beneficial in acute responses to stress and damage, the persistent accumulation of senescent cells is associated with many chronic diseases through their proinflammatory senescence-associated secretory phenotype (SASP). SASP expression is linked to DNA damage; however, the mechanisms that control the SASP are incompletely understood. More recently, it has been shown that senescent cells shed fragments of nuclear chromatin into the cytoplasm, so called cytoplasmic chromatin fragments (CCF). Here, we provide an overview of the current evidence linking DNA damage to the SASP through the formation of CCF. We describe mechanisms of CCF generation and their functional role in senescent cells, with emphasis on therapeutic potential.

## Introduction

Cellular senescence, a stable cell cycle arrest, is considered a hallmark of aging and contributes to aging-associated diseases (Herranz and Gil, 2018). While senescence acts acutely as a tumor suppressor mechanism, chronically it has a detrimental role in aging and associated pathologies (Childs et al., 2015). The pro-aging feature of senescence is in part mediated by the secretion of a large array of proinflammatory factors, termed the senescence-associated secretory phenotype (SASP), that leads to chronic inflammation, promoting tissue dysfunction and diseases (Rodier and Campisi, 2011). While the biological significance of the SASP has been intensively studied (Acosta et al., 2008; Coppé et al., 2010; Coppé et al., 2008; Krtolica et al., 2001; Kuilman et al., 2008), the mechanisms that initiate and sustain the SASP are poorly understood. Recently, we and others showed that SASP is mainly driven by the presence of cytoplasmic chromatin fragments (CCF), generated via a nuclear-cytoplasmic blebbing process (Dou et al., 2015; Dou et al., 2017; Glück et al., 2017; Ivanov et al., 2013; Vizioli et al., 2020; Yang et al., 2017). CCF possess a characteristic set of histone modifications and associated proteins. For example, CCF are enriched in heterochromatin markers including H3K9me3 and H3K27me3, but are devoid of certain euchromatin markers, such as H3K9ac. They are positive for the DNA damage marker γH2AX, but lack its usual partner, 53BP1 (Ivanov et al., 2013). Cytoplasmic DNA can also originate from several other sources such as micronuclei arising from chromosome segregation errors (Harding et al., 2017; Mackenzie et al., 2017; Terradas et al., 2010; Zhang et al., 2015), mitochondrial DNA (mtDNA) released from stressed mitochondria (West and Shadel, 2017), or cDNA produced by reactivation and high transcription levels of long-interspersed element-1 (LINE-1) (De Cecco et al., 2019; Simon et al., 2019). Accumulation of DNA in the cytoplasm serves as a potent danger signal that activates the innate immunity cytosolic DNA sensing cyclic GMP–AMP synthase (cGAS)-STING pathway, leading to proinflammatory responses (De Cecco et al., 2019; Dou et al., 2017; Glück et al., 2017; Harding et al., 2017; Mackenzie et al., 2017; Simon et al., 2019; West and Shadel, 2017; Yang et al., 2017). Here, we review the functional role of CCF as mediators of inflammation and associated pathologies with a focus on new insights into the origins and mechanisms that initiate and regulate the formation of CCF. We also discuss their translational relevance for identifying new targets and developing rationale strategies for therapeutic benefit.

## Formation of CCF

**Figure 1.**
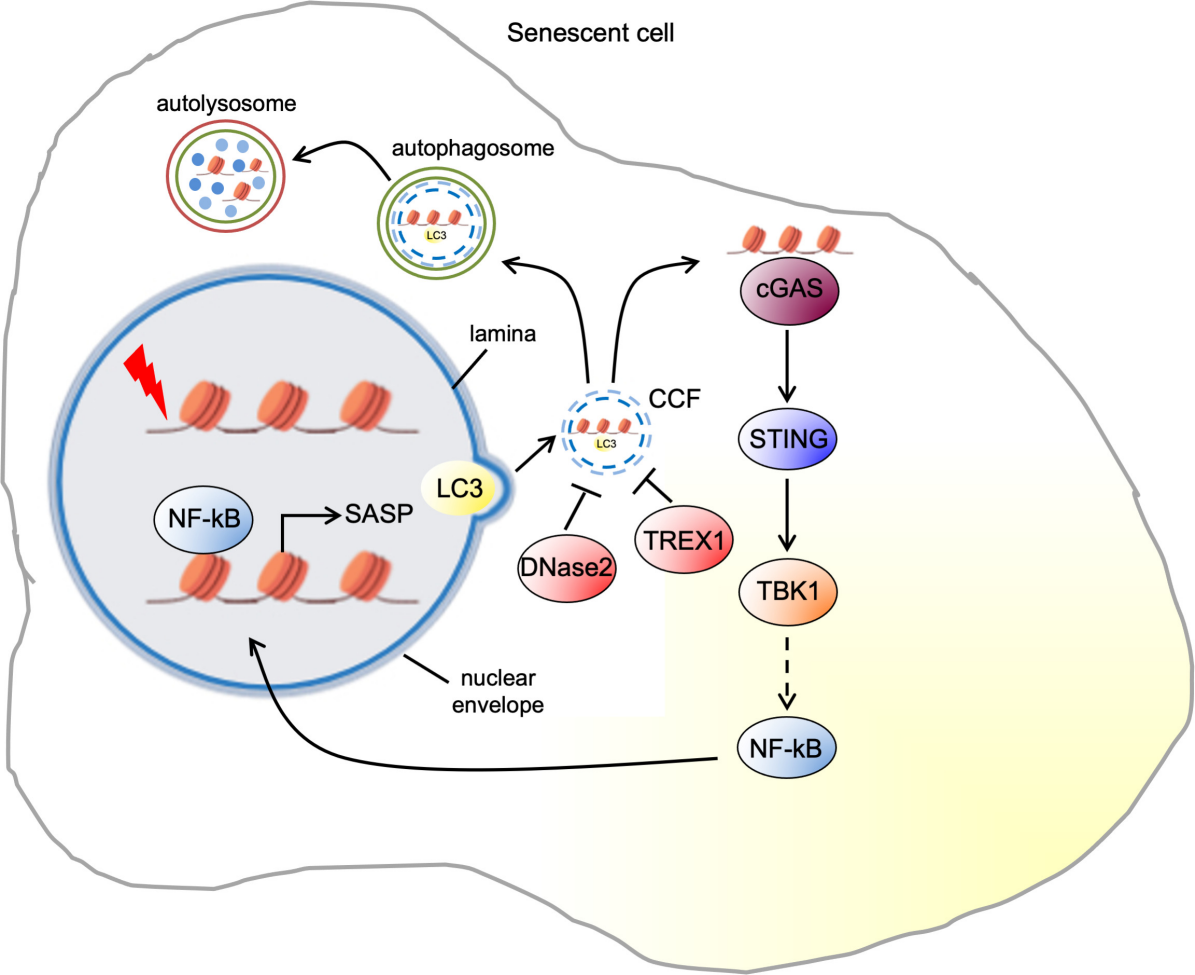
Pathways of cytoplasmic chromatin fragment formation and downstream signaling in senescent cells.

CCF are a type of cytoplasmic chromatin observed in multiple senescence models of primary human cells induced by DNA damage, oncogene activation, or replicative exhaustion. In addition, CCF have also been reported in several mouse models of senescence, such as liver tissue ectopically expressing oncogenic RAS, and hepatocytes exposed to stress caused by ionizing radiation (IR) or high-dose acetaminophen (Dou et al., 2017; Glück et al., 2017; Vizioli et al., 2020). Similar to micronuclei that are formed by abnormal chromosome segregation during mitosis, CCF accumulated during senescence can be large enough to be visualized by epifluorescence microscopy (up to 1–2 μM in diameter). However, unlike micronuclei, CCF are negative for lamin A/C and active chromatin marks (Ivanov et al., 2013). Live cell imaging revealed that CCF are generated by a nucleus-to-cytoplasm blebbing of chromatin through the nuclear membrane (Ivanov et al., 2013; Figure 1). This is in part thought to be mediated by the compromised integrity of nuclear lamina (Ivanov et al., 2013). The nuclear lamina is a fibrillar network underlying the inner nuclear membrane, composed of four nuclear lamins (lamin B1, B2, A, C) and associated proteins, where it serves to maintain nuclear stability and chromatin organization (Prokocimer et al., 2009). Lamins bind to chromatin at the lamina-associated domains (LADs). LADs are also associated with heterochromatic histone markers (Guelen et al., 2008). During cellular senescence there is a dramatic reorganization of chromatin, including the formation of senescence-associated heterochromatin foci (SAHFs) (Narita et al., 2003). At least as shown in oncogene-induced senescence, cells undergo a dramatic change in genome-nuclear lamina interactions with loss of LADs from the nuclear periphery and the formation of internal SAHFs (Lenain et al., 2017). Downregulation of lamin B1 in senescent cells is a trigger for SAHF formation (Sadaie et al., 2013). Another study also suggests that the increased density of nuclear pores that control the transport between the nucleus and cytoplasm is responsible for the repulsion of heterochromatin from the nuclear periphery and its reorganization into SAHFs (Boumendil et al., 2019). Nuclear lamina alterations appear to have a causative role in activating age-associated cellular responses (Serebryannyy and Misteli, 2018). For example, lamin A mutations are the cause of Hutchinson-Gilford progeria syndrome (HGPS) and cultured cells from these patients exhibit nuclear malformation, envelope abnormalities, and senesce prematurely in vitro (Goldman et al., 2004). Interestingly, upon induction of senescence, lamin B1 is downregulated, considered as a marker for cell senescence, while the expression of lamin A/B2/C is largely unaffected (Freund et al., 2012; Shimi et al., 2011). In HRasV12-induced senescent human diploid fibroblasts (HDF), the decrease of lamin B1 is mediated by activation of pRb pathway, since the expression of the adenovirus E1A oncoprotein is able to rescue the loss of lamin B1 (Shimi et al., 2011). More recently, it has been shown that the reduction of lamin B1 is mediated by autophagic degradation through an interaction of lamin B1 with the autophagy adaptor LC3 in HRASV12 senescent cells lung fibroblasts (IMR90). LC3-lamin B1, together with the associated chromatin regions, are transported from the nucleus to the cytoplasm leading to the formation of CCF (Dou et al., 2015; Figure 1). Importantly, when autophagy is inhibited or the interaction between LC3-lamin B1 blocked, the activated RAS-induced lamin B1 loss is prevented and oncogene-induced senescence is attenuated (Dou et al., 2015). These observations support a model in which lamin B1 is degraded via CCF, leading to loss of nuclear membrane integrity. Therefore, the exact role of nuclear membrane integrity in CCF formation remains incompletely understood.

The upstream signaling events that trigger the formation of CCF have been recently described. Accumulation of dysfunctional mitochondria increases the production of reactive oxygen species (ROS) in IR-induced senescent IMR90. ROS promote the activation of c-Jun N-terminal kinase (JNK) leading to CCF formation via a retrograde signaling pathway. Clearance of mitochondria and treatment with either a mitochondria-targeting antioxidant or a JNK inhibitor suppresses CCF formation in IR senescent cells (Vizioli et al., 2020). Downstream targets of JNK, as well as nuclear events that regulate CCF formation, are poorly understood and are significant questions for further exploration. However, it has been shown that in IR senescent cells JNK interacts with 53BP1, a nuclear protein involved in DNA damage repair. 53BP1 is excluded from CCF and is a negative regulator of CCF formation. While knockdown of 53BP1 leads to elevated CCF, its ectopic expression suppresses the induction of CCF (Vizioli et al., 2020). However, how 53BP1 inhibits CCF formation is still unclear. One possibility is that 53BP1-mediated suppression of double-strand break (DSB) end resection may be responsible for inhibition of CCF formation. Consistent with this idea, Mirin, a small molecule inhibitor of the nuclease MRE11 responsible for end resection, also inhibits CCF formation. Accordingly, formation of CCF may depend on DSB end resection by MRE11 (Vizioli et al., 2020). Future studies are needed to decipher the specific regulation of 53BP1 by JNK and the role of end resection in CCF formation.

In the cytoplasm, CCF can be degraded by the autophagosome-lysosome machinery (Figure 1). Therefore, it has been reported that the impaired autolysosomal function contributes to CCF accumulation. This is mainly mediated by the downregulation of DNase 2α, the unique endonuclease in the lysosomes, resulting in a decreased DNA degradation capacity of autolysosomes. Consistently, restoration of autolysosomal function with metformin or rapamycin treatment reduces CCF accumulation in oxidative stress-induced senescence (Han et al., 2020). Another work also reported that in HRasV12-induced senescent HDF, two cytoplasmic DNase enzymes DNase2 and TREX1 can remove CCF before they accumulate. However, in senescent cells, the expression of DNase2 and Trex1 is markedly reduced, resulting in the accumulation of CCF (Figure 1). The reduction of E2F transcriptional activity mediated by p16-RB pathway seems to be critical for the downregulation of DNase2 and TREX1 (Takahashi et al., 2018).

## CCF in signaling

Cytoplasmic DNA, typically a hallmark of pathogen infection, activates the cytosolic DNA-sensing cGAS-STING pathway that drives the type I interferon (IFN) pathway and proinflammatory cytokines to induce the innate immune response against the invading pathogens (Ablasser and Chen, 2019). However, CCF induce the SASP, also through the cGAS-STING pathway in all forms of senescence (Dou et al., 2017; Glück et al., 2017; Yang et al., 2017; Figure 1). CCF are recognized by the DNA sensor cGAS, which converts adenosine 5' triphosphate (ATP) and guanosine 5' triphosphate (GTP) into cyclic GMP-AMP (cGAMP) upon DNA binding (Motwani et al., 2019). cGAS colocalizes with CCF, and elevated cGAMP levels are detected in senescent cells, suggesting that CCF activate cGAS (Dou et al., 2017; Glück et al., 2017; Yang et al., 2017). Although cGAS senses double stranded DNA (dsDNA) in a sequence-independent manner (Li et al., 2013), the molecular mechanisms by which cGAS recognizes CCF are poorly understood. Recent work has suggested that the recognition of CCF by cGAS is potentiated by CCF-bound topoisomerase one cleavage complex (TOP1cc) in a dsDNA-dependent manner in HRasV12 or etoposide-induced senescent IMR90 (Zhao et al., 2020b). Chromatin architectural non-histone DNA-binding protein high-mobility group Box 2 (HMGB2) stabilizes the interaction between cGAS and TOP1cc (Zhao et al., 2020a). The identification of specific features of CCF that activate cGAS is a fundamental step to better understand the communication between cytoplasmic chromatin and innate immunity machinery.

Once generated by activated cGAS, cGAMP acts as second messenger that binds to and activates stimulator of IFN genes (STING), an adapter protein on the endoplasmic reticulum (ER) membrane (Motwani et al., 2019). Upon cGAMP binding, STING undergoes extensive conformational changes and recruits tank-binding kinase 1 (TBK1) which phosphorylates the transcription factors NF-κB and IRF3 (Motwani et al., 2019). Phosphorylated NF-κB and IRF3 enter the nucleus, to induce SASP and type I IFN responses, respectively (Zhang et al., 2019). In non-senescent cell models, it was reported that TBK1 phosphorylates the transcription factor NF-κB (p65) at S536 (Buss et al., 2004), which leads to enhanced transcriptional activation of NF-κB (Chen et al., 2005). Phosphorylation of the NF-κB inhibitor protein IκB by the IκB kinases (IKK), α and β, is also crucial for NF-κB nuclear translocation. TBK1 is also critical for the full activation of IKKβ, the upstream kinase of IκBα (Li et al., 2013) in a cGAS-STING-TBK1-TAK1-dependent manner (Balka et al., 2020). Probably, in senescent cells, CCF-cGAS-STING pathway-induced TBK1 activation mediates the activation of IKKβ which is required for the degradation of the IκB. This ultimately leads to the NF-κB nuclear translocation and SASP expression. Intriguingly, cGAS activation triggered by CCF does not induce type I INF production in HRasV12 or etoposide-induced early senescent IMR90, possibly due to activation of p38MAPK, which is known to inhibit STING-dependent IFN induction (Chen et al., 2017). Consistent with this hypothesis, p38 inhibition potentiated IFN-β in these cells (Dou et al., 2017). In contrast, retrotransposable LINE-1 elements (L1s) become transcriptionally derepressed in deeply senescent cells and activate a type I IFN response in a cGAS-dependent manner (De Cecco et al., 2019). In another study, CCF-induced cGAS activation leads to type I INF production in mouse embryonic fibroblasts undergoing to senescence by oxidative stress (Glück et al., 2017). These discrepancies might be due to different cell types, senescence stimuli, and timing used in these two studies. This will pave the way for future studies of IFN versus SASP initiated by cGAS-STING pathway. Dampened SASP factor levels in IR-induced senescent liver and lung in STING-deficient mice support the central role of STING-mediated NF-κB in SASP induction (Dou et al., 2017; Glück et al., 2017). However, future studies are needed to fully elucidate how the signal is transmitted from STING to activate NF-kB in senescence.

## CCF in disease

There is building evidence for the role of cytoplasmic DNA in age-associated inflammation in several diseases, both in mouse models and in humans. Several premature aging syndromes such as ataxia telangiectasia (AT) and HGPS disease are accompanied by the release of genomic DNA into the cytoplasm (Lan et al., 2019). AT is a severe neurodegenerative syndrome which is caused by gene defects in ATM, a large PI3K family protein kinase that is prominently involved in DNA DSB repair (Song et al., 2019). Accumulation of γ‐H2AX-enriched cytoplasmic DNA in the form of nuclear buds, speckles, and large fragments was detected in AT and HGPS patient skin fibroblasts, along with the expression of inflammatory genes, including IFN‐α, TNF‐α, and IL‐6 (Lan et al., 2019). ATM deficiency also induced accumulation of cytosolic DNA in mouse microglia which primes them for a proinflammatory response in a cGAS-STING-dependent manner (Song et al., 2019). Similar observations were also found in HGPS, which is a premature aging syndrome caused by mutations in the nuclear envelope structural protein lamin A (LMNA). LMNA mutations cause loss of nuclear integrity leading to accumulation of cytosolic DNA. This accumulation is linked to inflammation, for example, increased IL‐6 signaling in HGPS cells (Kreienkamp et al., 2018; Lan et al., 2019).

## Perspective

**Figure 2.**
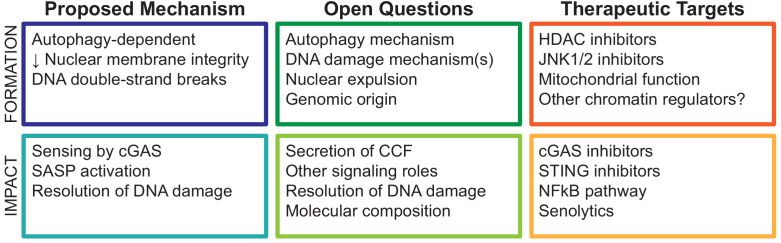
Conceptual summary of cytoplasmic chromatin fragment formation and function.

Persistent DNA damage response (DDR) signaling is essential to establish and maintain the SASP in senescent cells. However, the mechanisms linking DDR and SASP are incompletely explored (Meyer et al., 2017; Rodier and Campisi, 2011). CCF connect these central concepts in senescence, and the pathways associated with CCF formation and function have exciting therapeutic potential. Many important questions remain, particularly the regulatory mechanisms governing the assembly and expulsion of damaged genomic DNA as CCF and the functional consequences of CCF formation beyond the SASP (Figure 2).

Formation of CCF is linked to the DDR and DNA repair, but the mechanisms are unclear. There are two major DNA DSB repair pathways, nonhomologous end joining (NHEJ) and homologous repair (HR). NHEJ is faster and error prone compared to HR, which involves single-strand resection of DNA ends, a search for homology, and resolution by templated extension of the broken region, gap filling, and ligation (Scully et al., 2019). Because faithful repair by HR requires a sister chromatid template in late S or G2 phases of the cell cycle, the choice between these pathways is carefully controlled by several factors, including the DNA repair scaffold protein 53BP1 (Mirman et al., 2020). 53BP1 is a suppressor of HR repair, in part by limiting DSB single-stranded end resection (Bunting et al., 2010). Interestingly, 53BP1 suppresses CCF formation, suggesting that DSB end resection is required for CCF formation (Fink et al., 2011; Vizioli et al., 2020). Senescent cells have reduced expression of most DNA repair factors and are known to be defective in both NHEJ and HR repair (Mao et al., 2012; Seluanov et al., 2004). Taken together, this suggests that an alternative function of CCF formation may be resolution of end-resected DSB in senescent cells (Lan et al., 2014), given that CCF are highly enriched in γH2AX and, by implication, DSBs. However, it is unknown whether other functions of 53BP1 and MRE11 also play a role, which other DNA repair factors are involved, and the role of canonical 53BP1-associated DDR mediators such as ATM and ATR. An additional unaddressed question is whether the DDR drives SASP exclusively through CCF formation. Supporting the possibility of multiple pathways linking DDR to SASP, ATM has been shown to phosphorylate NF-κB pathway regulator NEMO in response to acute DNA damage, as well as senescence contexts, activating NF-κB (Wu et al., 2006; Zhao et al., 2020b). Lastly, it is unclear whether cytoplasmic localization of γH2AX and other chromatin-associated proteins has additional signaling roles in senescent cells beyond cGAS-STING pathway activation. To this end, studies profiling the molecular composition of CCF are beginning to emerge (Zhao et al., 2020a), and data on the genomic origin of CCF will be of particular benefit to this emerging field.

Regarding therapeutic potential, the CCF-SASP pathway is a novel advance in cell senescence with wide potential application in treatment of age-associated diseases to which the SASP is causatively linked (Georgilis et al., 2018; He and Sharpless, 2017; van Deursen, 2014). Inhibition or modulation of the SASP using so-called senomorphic treatments provides an alternative approach to treatment of these diseases, as compared to complete removal of senescent cells using senolytic treatments. Indeed, senolytics are known to have toxic effects in humans (Kaefer et al., 2014; van Deursen, 2019; Wilson et al., 2010), potentially linked to off-target effects on non-senescent cells, or linked to emerging beneficial roles of senescent cells in normal physiology. Approaches to reduce or alter the SASP have shown promise in animal models (Milanovic et al., 2018; Yoshimoto et al., 2013), suggesting that novel therapeutic targets could greatly advance this area of research. As mentioned above, there are early candidates that prevent CCF formation and SASP in senescent cells (Chapman et al., 2019; Vizioli et al., 2020). Upstream of CCF formation, regulation of mitochondrial ROS, and downstream signaling pathways are beginning to emerge as important targets: the HDACi trichostatin A and SAHA prevent CCF formation by reducing mtROS through an indirect mechanism (also *in vivo*), and the JNKi SP600125 prevents CCF formation by blocking JNK1/2 activation downstream of mitochondrial ROS (Vizioli et al., 2020). Further clarification of mito-nuclear signaling pathways as well as the nuclear autophagy-dependent mechanism of CCF formation can also identify new targets with therapeutic potential. Downstream of CCF formation, it will be important to clarify the mechanism of cGAS recognition of CCF, as well as potential roles of other DNA sensors. Speculative functions of CCF beyond control of the SASP may also have therapeutic value, including resolution of DNA damage, autophagic CCF degradation, and CCF secretion from the cell.

In summary, the study of CCF formation and function has provided novel advances in our understanding of nuclear autophagy, genome integrity, and senescence-associated inflammation. This work contributes to a growing field in biomedical research, the study of cytoplasmic DNA species, and their role in human disease. By further unraveling the mechanisms involved, this work can advance our understanding of age-associated disease vulnerability and therapies to delay this process and improve human health.
